# 

**Published:** 1884-06

**Authors:** 


### CHICAGO MEDICAL JOURNAL & EXAMINER.

EDITORS:
JAMES NEVINS HYDE, A.M., M.D.,
HAROLD N. MOYER, M.D.,
W. W. JAGGARD, A.M., M.D.

CHICAGO MEDICAL PRESS ASSOCIATION.

AUG. 2D

1875.

CHICAGO:
*PUBLISHED BY THE CHICAGO MEDICAL PRESS ASSOCIATION*
193 W. MADISON STREET.

☞ Read the Notice of this Journal among Advertisements

